# A health literacy analysis of the consumer-oriented COVID-19 information produced by ten state health departments

**DOI:** 10.5195/jmla.2021.1165

**Published:** 2021-07-01

**Authors:** Nandita S. Mani, Terri Ottosen, Megan Fratta, Fei Yu

**Affiliations:** 1 nanditam@unc.edu, Associate University Librarian for Health Sciences, Director of the Health Sciences Library, University of North Carolina at Chapel Hill, Chapel Hill, NC; 2 ottosen@email.unc.edu, Community Engagement and Health Literacy Librarian, Health Sciences Library, University of North Carolina at Chapel Hill, Chapel Hill, NC; 3 mfratta@email.unc.edu, Community Outreach & Global Health Librarian, Health Sciences Library, University of North Carolina at Chapel Hill, Chapel Hill, NC; 4 feifei@email.unc.edu, Health Informatics Librarian, Assistant Professor, Health Sciences Library, School of Information & Library Science, University of North Carolina at Chapel Hill, Chapel Hill, NC

**Keywords:** health literacy, communication, information design, COVID-19, public health, consumers, health information, infodemic, state health department, health education, health communication

## Abstract

**Objective:**

The COVID-19 pandemic highlights the public's need for quality health information that is understandable. This study aimed to identify (1) the extent to which COVID-19 messaging by state public health departments is understandable, actionable, and clear; (2) whether materials produced by public health departments are easily readable; (3) relationships between material type and understandability, actionability, clarity, and reading grade level; and (4) potential strategies to improve public health messaging around COVID-19.

**Methods:**

Based on US Centers for Disease Control and Prevention statistics from June 30, 2020, we identified the ten states with the most COVID-19 cases and selected forty-two materials (i.e., webpages, infographics, and videos) related to COVID-19 prevention according to predefined eligibility criteria. We applied three validated health literacy tools (i.e., Patient Education Materials Assessment Tool, CDC Clear Communication Index, and Flesch-Kincaid Grade Level) to assess material understandability, actionability, clarity, and readability. We also analyzed correlations between scores on the three health literacy tools and material types.

**Results:**

Overall, COVID-19 materials had high understandability and actionability but could be improved in terms of clarity and readability. Material type was significantly correlated with understandability, actionability, and clarity. Infographics and videos received higher scores on all tools.

**Conclusions:**

Based on our findings, we recommend public health entities apply a combination of these tools when developing health information materials to improve their understandability, actionability, and clarity. We also recommend using infographics and videos when possible, taking a human-centered approach to information design, and providing multiple modes and platforms for information delivery.

## INTRODUCTION

Since its emergence in December 2019, the COVID-19 pandemic has had far-reaching implications for global public health, one of which is the need for quality health information that is understandable by the public. The World Health Organization (WHO) declared in February 2020 that the current global health crisis is also an information crisis, or infodemic [1]. The WHO defines the current infodemic as an overabundance of information, both accurate and inaccurate, “that makes it hard for people to find trustworthy sources and reliable guidance when they need it” [2]. While greater access to health information exists, the overwhelming supply and questionable nature of the available information makes it difficult for the consumer to navigate, understand, and act upon [3].

The infodemic highlights the role that health literacy plays in information consumption. Health literacy enables people to effectively act on information to make appropriate health care decisions and lead healthier lives. Although the ability to read is an important component of health literacy, it encompasses a wide variety of skills, including numeracy, critical analysis, and communication and interaction skills [4, 5]. It is estimated that approximately ninety million people, nearly half of all adults in the US, have difficulty understanding and acting upon health information [6]. Despite the fact that most US adults read at an eighth-grade level or below, most health care materials are written at a tenth-grade level [7]. If people cannot read and understand health information, they may be less likely to act or change their behavior. Health information must be actionable and appropriate for people with a wide range of health literacy skills. It must also be simple and straightforward, particularly for health messages in times of urgency or during a health crisis [8]. During the public health challenge of the COVID-19 pandemic and the resulting infodemic, health literacy and clear health communication are essential determinants of health status and healthy behavior [9].

Experts have called for the application of health literacy principles to the creation of COVID-19 messaging, and health literacy should be considered as a component of the COVID-19 public health response framework [10–12]. As much of the COVID-19 public health messaging focuses on effecting behavior change to promote disease prevention such as hand washing, social distancing, and wearing face masks [10], it is imperative that these messages be crafted in an easily understandable and actionable manner [11, 13, 14].

Only a few studies have assessed the health literacy of COVID-19 public health messaging and have reported concerning results. One study found that the readability of online COVID-19 information provided by the WHO, the US Centers for Disease Control and Prevention (CDC), and the governments of fifteen countries exceeded the sixth-grade reading level as recommended by the American Medical Association for patient education materials [15, 16]. This study also found that COVID-19 information on 137 webpages from US federal and state sources averaged over the eleventh-grade reading level. Another study assessed COVID-19 information for the public retrieved by search engines and identified deficiencies in the readability, understandability, and actionability of the assessed materials [8].

Each state in the US is handling the health crisis with varied policies, strategies, and safety guidelines [17], which can potentially result in varied content and formats of public health messaging. We focused on the ten states with the highest number of cases, as the public health messaging around prevention in those states may have the largest impact on reducing future infection rates. Since the CDC has publicized three ways to slow the spread of the virus—wear a mask, stay at least six feet away from others, and wash hands often—public health messaging on these prevention measures could help alter the course of the pandemic [18]. The extent to which the public health messaging created by states is understandable, actionable, clear, or easy to read is unknown. Therefore, the objective of this study was to systematically examine and assess COVID-19 health information materials created by state public health departments in the US. Our study aimed to identify (1) the extent to which COVID-19 messaging is understandable, actionable, and clear; (2) whether materials produced by public health departments meet the recommended reading grade level; (3) whether relationships exist between understandability, actionability, clear communication, reading grade level, and material type; and (4) potential strategies to improve creation of public health messaging around COVID-19.

## METHODS

### Sample selection and inclusion criteria

We identified the ten states with the highest number of COVID-19 cases based on CDC-reported cases in the US on June 30, 2020 [19] and reviewed public health messaging extracted directly from those states' public health department websites that are accessible to the general public. Two reviewers (NM, TO) visited the ten states' public health department websites and reviewed and screened all COVID-19-related materials. Materials were included in the study if they were (1) written or presented in English and (2) about COVID-19 on the topics of handwashing, mask wearing, social distancing, or prevention in general. Materials were excluded if their topics were not about COVID-19 prevention. Material types that we included were webpages (including PDFs), infographics, and videos. For each included material, an identification number was manually assigned that started with the state acronym, such as CA-1 or NY-3. The following attributes of all included materials were recorded in a Microsoft Excel spreadsheet (Appendix A) and used to facilitate the assessment process: material identification number, material title, material type, and material URL.

### Scoring tools and interpretation

We applied three validated health literacy tools to assess understandability, actionability, clarity, and readability of materials created by state public health departments. The Patient Education Materials Assessment Tool (PEMAT) was used to evaluate and compare the understandability and actionability of the materials [20], the CDC Clear Communication Index (Index) was applied to assess the clarity of the materials [21], and the Flesch-Kincaid Grade Level (FKGL) score was calculated to determine the readability of the materials [22]. All three tools have been used previously to assess consumer and patient health documents [8, 23–26]. Using this combination of tools provides a multifaceted means of assessing the state health department COVID-19 documents and allows for a holistic evaluation [23, 25].

### Understandability and actionability

The PEMAT for Printable Materials (PEMAT-P) was applied to printable materials such as webpages (including PDFs) and infographics, whereas the PEMAT for Audiovisual Materials (PEMAT-A/V) was applied to videos [20]. The PEMAT-P consists of seventeen items measuring understandability and seven items measuring actionability, whereas the PEMAT-A/V has thirteen items measuring understandability and four items measuring actionability. Understandability items in the PEMAT determine whether readers with varying backgrounds and levels of health literacy skills can process and explain key messages. The understandability is assessed upon content, word choice and style, use of numbers, organization, layout and design, and use of visual aids. Actionability items appraise whether readers will know how to use or apply the information presented. In accordance with the PEMAT User's Guide [20], two reviewers (NM, TO) independently applied the appropriate PEMAT tool (PEMAT-P or PEMAT-A/V) to the materials using the PEMAT Auto-Scoring Form [27]. For each of the twenty-four (PEMAT-P) or seventeen (PEMAT-A/V) PEMAT items, reviewers assigned a score of a 0/Disagree, 1/Agree, or Not Applicable. Once independent scoring of all items was complete, both reviewers met and discussed inconsistencies in their scores until discrepancies were resolved and consensus was achieved. The PEMAT Auto Scoring Form (https://www.ahrq.gov/sites/default/files/publications/files/pemat_form.xls) generated percentage scores for the understandability and actionability for each material, as explained in the PEMAT User's Guide. The higher the score, the more understandable or actionable the materials are regarded [20]. A score of 70% has been used as a benchmark for materials to be considered highly understandable or actionable, with a score below 70% indicating poor understandability or actionability [23, 28, 29]. This process was previously used to evaluate COVID-19 consumer health materials using the PEMAT [8].

### Clarity

The Index is a validated tool that provides a set of research-based criteria to develop, improve, and assess public communication products [21]. The Index can be used both to inform the design and development of new communications and to assess the clarity and usability of existing messages. As a clear communication tool, it addresses health, science, and risk communication and reduces subjectivity in evaluating materials [30]. The Index evaluates materials in seven areas: main message and call to action, language, information design, state of the science, behavioral recommendations, numbers, and risk [31]. We applied the Full Index Score Sheet (Index-Full, https://www.cdc.gov/ccindex/pdf/full-index-score-sheet.pdf) to print materials (i.e., webpages) and the Modified Index Score Sheet (Index-Mod, https://www.cdc.gov/ccindex/pdf/modified-index-scoresheet.pdf) to short form and oral communications (i.e., infographics and videos). The Index-Full has twenty research-based items, and the Index-Mod has thirteen items. In accordance with the Index User Guide [21], two reviewers (NM, TO) independently applied the Index tool to the materials using either Index-Full or Index-Mod. Each material received a numerical score of either 0/No or 1/Yes for each item. Per the Index User's Guide, individual scores for each material were converted to an overall score on a scale of 100, where 90 or higher is considered passing or “easy to read” [21, 23, 24].

### Readability

The FKGL score [22] is one of the most commonly used readability measures for patient education materials [32–36] and online consumer health information [37–39]. The FKGL score reports readability as a grade equivalent reading level [16]. The score is calculated using a mathematical formula based on two factors: 1) sentence length—average number of words in a sentence, and 2) word length—average number of syllables in a word. The rationale behind the score is that longer sentences are more difficult to understand than shorter sentences, and, likewise, words with more syllables are harder to read than words with fewer syllables. Materials containing fewer than one hundred words and videos were excluded from calculating a FKGL score, as texts of fewer than one hundred words may produce invalid readability results [8, 40]. One reviewer (TO) generated the FKGL score for materials using the library's Readable Pro subscription [41]. Readable Pro does not require document cleaning such as removing formatting, and documents could be directly uploaded for assessment. We classified materials at or below the sixth-grade level as “easy,” those from seventh- to ninth-grade as “average,” and those at the tenth-grade level or higher as “difficult” [8].

### Statistical analysis

All statistical analyses were conducted using SPSS [42]. The statistical measures produced and tested included (1) interrater reliability (IRR) by Cohen's kappa [23, 43] using the two reviewers' independent scores before they reached consensus; (2) the mean, median, standard deviation (SD), interquartile range (IQR), minimum, maximum, and frequency scores associated with PEMAT, Index, and FKGL; and (3) statistical significance of the correlations between material type and PEMAT, Index, and FKGL scores. Material types were coded by the intensity of visual cues as 1=web page, 2=infographic, and 3=video. Both Pearson and Spearman correlation tests were two-sided [43, 44].

## RESULTS

A total of 42 materials from 10 US state health departments (Appendix A) were reviewed and evaluated for this study. The average number of materials from each state was 4.2, with a range of 3 to 7. Nineteen of the materials were webpages (including PDFs), 19 of the materials were infographics, and 4 were videos. The IRR between the two reviewers was K=0.941 for PEMAT and K=0.942 for Index.

### PEMAT

The PEMAT-P was applied to 38 materials (i.e., webpages and infographics), and the PEMAT-A/V was applied to 4 materials (i.e., videos). Each material was assessed for understandability and actionability as per the PEMAT users' guide.

### Understandability

**Figure 1:**
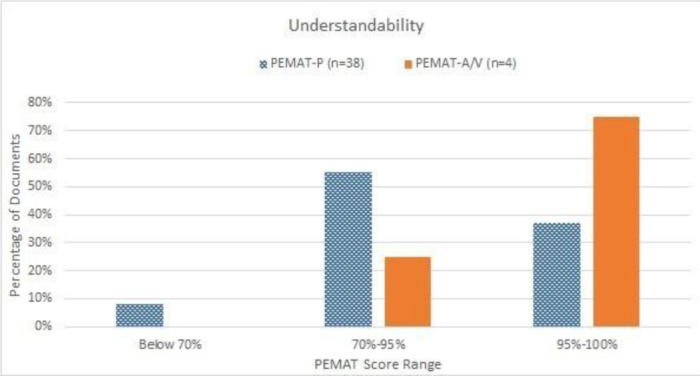
Distribution of materials by understandability assessment using PEMAT. A score of 70% or above is considered highly understandable, and a score below 70% indicates poor understandability

The average of all reviewed materials assessed by either PEMAT-P or PEMAT-A/V for understandability was 88.67% (SD±17.69%), with a range between 21% and 100% (Table 1). Most (92%) materials reviewed by PEMAT-P had an understandability score above 70%, and all materials reviewed by PEMAT-A/V had an understandability score above 70% (Figure 1).

**Table 1:** Understandability, actionability, communication clarity, and readability of public materials by the Dept. of Health of 10 US state governments

Health Literacy Tool	# Materials	Mean (SD)	Median (IQR)	Minimum	Maximum
PEMAT-P Understandability	38	88.18% (±18.16%)	94% (±14.75%)	21%	100%
PEMAT-P Actionability	38	87.26% (±14.51%)	83% (±20%)	40%	100%
PEMAT-A/V Understandability	4	93.25% (±13.50%)	100% (±20.25%)	73%	100%
PEMAT-A/V Actionability	4	100% with (±0%)	100% (±0%)	100%	100%
PEMAT-All Understandability	42	88.67% (±17.69%)	94% (±14.75%)	21%	100%
PEMAT-All Actionability	42	88.48% (±14.30%)	100% (±20.25%)	40%	100%
Index-Full	19	73.57 (±14.22)	73.7 (±11.6)	50	100
Index-Mod	23	82.23 (±10.74)	81.8 (±15.90)	58.3	100
Index-All	42	78.32 (±13.03)	78.35 (±17.27)	50	100
FKGL	34	7.11 (±2.60)	7.30 (±3.5)	1.7	12.5

More than 50% of reviewed materials were rated “agree” for all measures in the PEMAT understandability items (Appendix B). Of note, all materials were rated “agree” for “The material makes its purpose completely evident” (item 1) and “The material does not expect the user to perform calculations” (item 7). However, 39% of materials were rated “disagree” for “The material uses visual aids whenever they could make content more easily understood” (item 15). In addition, 21% of materials were rated “disagree” for “The material does not include information or content that distracts from its purpose” (item 2).

### Actionability

**Figure 2:**
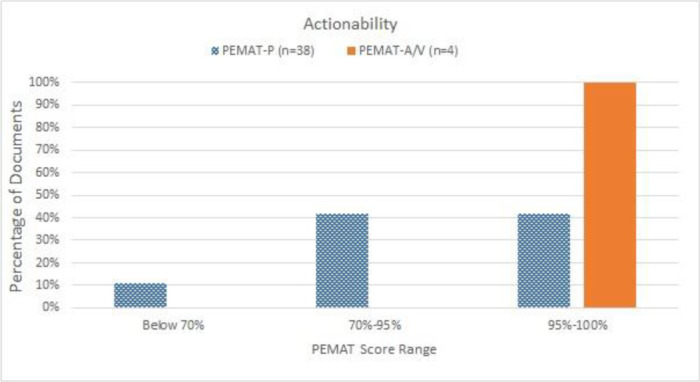
Distribution of materials upon actionability assessment using PEMAT. A score of 70% or above is considered highly actionable, and a score below 70% indicates poor understandability

The average of all materials assessed by either PEMAT-P or PEMAT-A/V for actionability was 88.48% (SD ±14.30%), with a range between 40% and 100% (Table 1). Most (89%) of the materials assessed by PEMAT-P had actionability scores above 70%, and all materials assessed by PEMAT-A/V had actionability scores of 100% (Figure 2). However, 39% of materials were rated “disagree” for “The material uses visual aids whenever they could make it easier to act on the instructions” (item 26).

### Index

**Figure 3:**
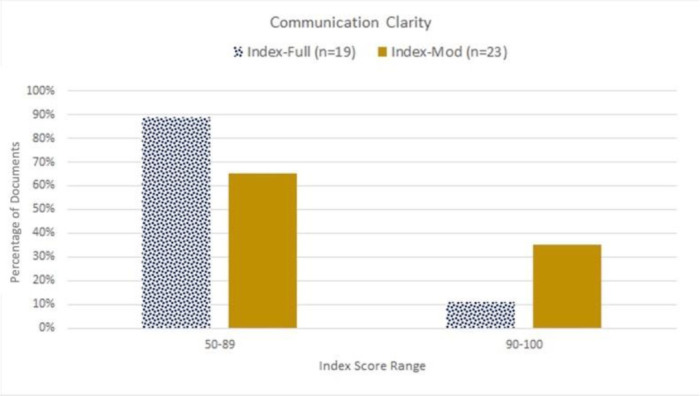
Clarity of communication materials as assessed by the Index. A score above 90% is considered passing or “easy to read”

The Index-Full was applied to 19 materials (i.e., webpages), and the Index-Mod was applied to 23 materials (i.e., infographics and videos). The average of all reviewed materials assessed by both Index-Full and Index-Mod was 78.32 (SD ±13.03), with a range between 50 and 100 (Table 1). Only 11% of the materials assessed by Index-Full had scores above 90, whereas 35% of materials assessed by Index-Mod had scores above 90 (Figure 3).

Assessed using either Index-Full or Index-Mod, more than 60% of materials were rated “Yes” for most index items (Appendix C). Of note, 100% of materials were rated “Yes” for contains call to action for the primary audience (item 5) and including one or more behavior recommendations for the primary audience (item 12) (Appendix B). However, 92% of materials were rated “disagree” for explaining what authorities know and do not know about the topic (item 11), and 43% of materials were rated “disagree” for addressing both risks and benefits of recommended behaviors (item 19).

### FKGL

**Figure 4:**
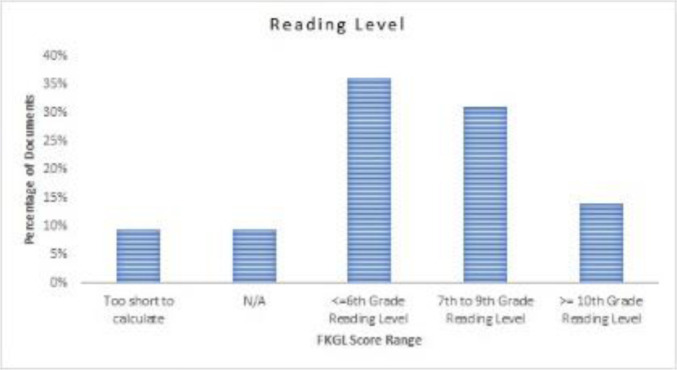
Distribution of materials by readability assessment using FKGL. Materials with a FKGL at or below the sixth-grade level are easy to read, seventh- to ninth-grade level are of average difficulty, and tenth-grade or higher are difficult to read

Among the 42 materials, 34 received FKGL scores; 4 materials were too short to calculate FKGL scores, and 4 were in video format, for which FKGL does not apply (Figure 4). The average FKGL score was 7.11 (SD ±2.60), with a range between 1.7 and 12.5 (Table 1). Thirty-six percent of materials had reading levels at or below the sixth-grade reading level, 31% had reading levels between the seventh- and ninth-grade level, and 14% were equal to or above the tenth-grade reading level (Figure 4).

### Relationships between assessment scores and material types

We identified statistically significant positive correlations between PEMAT understandability scores and actionability scores (n=42, Pearson r=0.486, Spearman's rho r=0.583, *p*<0.01) and between PEMAT understandability and actionability scores and Index scores (Pearson r=0.619, Spearman's rho r=0.567, *p*<0.01; Pearson r=0.511, Spearman's rho r=0.514, *p*<0.01, respectively). We also detected statistically significant negative correlations between PEMAT understandability and actionability scores and FKGL (Pearson r=−0.606, Spearman's rho r=−0.584, *p*<0.01; Pearson r=−0.525, Spearman's rho r=−0.591, *p*<0.01, respectively) and between Index scores and FKGL (Pearson r=−0.522, Spearman's rho r=−0.545, *p*<0.01).

We found statistically significant positive correlations between material type and PEMAT understandability and actionability scores (Pearson r=0.422, Spearman's rho r=0.601, *p*<0.01; Pearson r=0.557, Spearman's rho r=0.644, *p*<0.01 respectively) but a negative correlation between material type and FKGL (Pearson r=−0.672, Spearman's rho r=−.0.704, *p*<0.01). There was no statistically significant relationship between material type and Index scores (*p*>0.05). Therefore, video and infographic material types were associated with higher understandability and actionability and lower reading levels than webpages.

## DISCUSSION

This study used three health literacy assessment tools to gauge the understandability, actionability, clarity, and readability of COVID-19 prevention materials that are publicly accessible on ten US state health department websites. Overall, we found that COVID-19 health information materials provided by state governments had high understandability and actionability scores but needed improvement in the areas of clarity and readability.

The majority of the materials achieved a “passing” score in understandability and actionability, as measured by average PEMAT understandability and actionability scores above the benchmark of 70% [20, 23, 28]. While materials scored well overall on the PEMAT, the materials scored lowest on PEMAT items pertaining to the use of visual aids to make content easier to understand and act upon. Also, some materials contained information or content that distracted from their purpose. The materials evaluated in this study were lacking in communication clarity as evidenced by an average Index score below the passing score of 90 [21, 23, 24]. These findings are in accordance with previous studies in which health information materials did not achieve a passing Index score and could be revised for clarity [23–26]. The materials scored lowest on Index items pertaining to explaining what authorities know and do not know about the topic and the use of visual cues and visuals to emphasize main messages. Based on these weaknesses, the understandability, actionability, and clarity of the materials could be improved by incorporating visual cues or images to illustrate key points and removing extraneous or distracting information.

We found that a minority of materials reviewed were at or below a sixth-grade reading level, which is the reading level recommended by the American Medical Association for consumer health and patient education materials [16]. Additionally, 45% of the materials were rated at reading levels above the seventh-grade and are considered to be of either “average difficulty” or “difficult” to read [8, 45]. Our findings are consistent with Mishra and Dexter [15]. As 20% of Americans read at or below the fifth-grade level, public health departments should strive to write their COVID-19 materials at a more appropriate reading grade level [7].

We observed significant correlations among three types of health literacy assessment scores (i.e., PEMAT, Index, and FKGL). The positive correlation between PEMAT understandability and actionability suggests that improving the understandability of a material will lead to greater actionability, and vice versa. The positive correlations between PEMAT and Index scores suggest that enhancing understandability or actionability will help improve the communication clarity of materials, and vice versa. We also identified a correlation between a lower, more appropriate grade-level FKGL score and higher PEMAT understandability, PEMAT actionability, and Index scores, which is consistent with previous findings [43, 44]. Thus, materials written at a lower grade level correlate with higher understandability, actionability, and clarity. Additionally, infographics and videos were associated with higher understandability, actionability, and clarity scores than textual materials; infographics were also correlated with lower, more appropriate, FKGL scores.

Based on our findings, we propose the following strategies to improve the design and creation of public health messaging around COVID-19. First, using a combination of health literacy tools (i.e., PEMAT, Index, and FKGL) can aid in the development and revision of health messages and patient education materials. These health literacy tools will be most useful when they are not used in isolation [20, 45, 46]. Incorporating the PEMAT, Index, and FKGL tools into the material creation and review process will enhance the understandability, actionability, clarity, and readability of the materials [47], whereas using only one of these tools may serve to ignore other aspects of health literacy. Key items from the PEMAT to consider are using plain language, informative headers, and active voice; key Index items to incorporate are having one main message, using visual cues to emphasize the main message, and including a call to action. In addition, we recommend using free or proprietary readability assessment tools to revise materials and improve the grade-level readability.

Second, writing and revising materials through a health literacy lens is an especially important consideration for the dissemination of COVID-19 information to populations at high risk for limited health literacy such as older adults, people of color, and people with chronic health conditions, as they are also at higher risk for the effects of COVID-19 [8]. Librarians are familiar with their communities' health information literacy needs and can be a valuable member of public health teams working to improve the health of their communities [3]. Encouraging a human-centered approach, design teams should work with focus groups to test materials throughout the design and development phases of information creation prior to public consumption [48]. It is thus imperative to consider those at high risk for both low health literacy and severe illness from COVID-19 when forming focus groups to test these new health information materials.

Third, those developing health communication materials should consider presenting information in infographic or video form when possible. We found materials using these forms to be associated with higher understandability, actionability, and clarity than text-based materials. If the content does not lend itself to infographic or video form, incorporating visuals can help to reinforce the main message. This strategy aligns with Schubbe et al.'s findings that pictorial health information increased understanding for low health literacy populations [49]. Nevertheless, a digital divide still exists [50–52], and there are significant gaps in access to technology and Internet connectivity, which can hinder the public from accessing critical information. State governments should consider a variety of technological platforms (e.g., websites, social media, mobile apps) and information delivery modes, such as traditional direct-appeal public outreach (e.g., public radio, billboards, flyers, and handouts), to overcome potential information barriers.

While we reviewed COVID-19-related health information materials from ten US state government websites, our intention was not to make comparisons between individual materials or how the messaging differed between states. We focused on this sample because these materials were released from official health department sources and readily accessible to the general public to fight the COVID-19 pandemic. Future research is warranted to further examine the health information materials of a larger pool of US health information produced by official sources; to that end, attempts should also be made to evaluate public health messaging from an international lens. A significant limitation of this study is that only materials written in English were evaluated. Future research should include materials written in other languages to ensure adequate health literacy practices are in use and to assess the extent to which cultural context impacts health information design. Additionally, while our study makes a compelling case for the use of video-based health information materials, our overall sample size of video materials was small. Future research should be aimed at specifically elucidating the benefits of video-based materials.

In conclusion, this study examined the COVID-19 health information provided by state public health departments using a combination of health literacy assessment tools. We found the public health messaging assessed in this study to be understandable and actionable; however, there are many opportunities to improve clarity and readability. Our findings can help state public health departments enhance their public messaging related to COVID-19 prevention. In addition, we propose utilizing health literacy tools in the development of public health materials, taking a human-centered approach to information design, and providing multiple modes and platforms for information delivery. As can be seen with the infodemic surrounding the COVID-19 pandemic, the importance of crafting clear communication, especially in times of public health crisis, is evident.

## Data Availability

All data associated with our results are available in Appendix A.

## SUPPLEMENTAL FILES

**Appendix A** Material Review Scoring including PEMAT, Index, and FKGL

**Appendix B** PEMAT Measure Scoring

**Appendix C** Index Item Scoring
