# Supplementary material for: A health literacy analysis of the consumer-oriented COVID-19 information produced by ten state health departments
Source: J Med Libr Assoc. 2021 Jul 1;109(3):422–31. doi: 10.5195/jmla.2021.1165 (PMC8485956; doi:10.5195/jmla.2021.1165)
Supplement: Supplementary file 2 — Appendix B PEMAT Measure Scoring [file jmla-109-3-422-s02.docx]

Appendix B. PEMAT Measure Scoring

| **PEMAT Measures** | **Materials scored "Agree" (%)** | **Materials scored "Disagree" (%)** | **Materials scored "N/A" (%)** |
| --- | --- | --- | --- |
| **UNDERSTANDABILITY** |  |  |  |
| TOPIC: CONTENT |  |  |  |
| 1. The material makes its purpose completely evident | 100% | 0% | 0% |
| 2. The material does not include information or content that distracts from its purpose | 79% | 21% | 0% |
| TOPIC: WORD CHOICE & STYLE |  |  |  |
| 3. The material uses common, everyday language | 86% | 14% | 0% |
| 4. Medical terms are used only to familiarize audience with the terms. When used, medical terms are defined | 93% | 7% | 0% |
| 5. The material uses the active voice | 90% | 10% | 0% |
| TOPIC: USE OF NUMBERS |  |  |  |
| 6. Numbers appearing in the material are clear and easy to understand | 76% | 0% | 24% |
| 7. The material does not expect the user to perform calculations | 100% | 0% | 0% |
| TOPIC: ORGANIZATION |  |  |  |
| 8. The material breaks or “chunks” information into short sections | 90% | 5% | 5% |
| 9. The material’s sections have informative headers | 88% | 7% | 5% |
| 10. The material presents information in a logical sequence | 95% | 5% | 0% |
| 11. The material provides a summary | 81% | 10% | 10% |
| TOPIC: LAYOUT & DESIGN |  |  |  |
| 12. The material uses visual cues (e.g., arrows, boxes, bullets, bold, larger font, highlighting) to draw attention to key points | 88% | 10% | 2% |
| 13. Text on the screen is easy to read | 50% | 25% | 25% |
| 14. The material allows the user to hear the words clearly (e.g., not too fast, not garbled) | 75% | 25% | 0% |
| TOPIC: USE OF VISUAL AIDS |  |  |  |
| 15. The material uses visual aids whenever they could make content more easily understood (e.g., illustration of healthy portion size) | 61% | 39% | 0% |
| 16. The material’s visual aids reinforce rather than distract from the content | 61% | 3% | 37% |
| 17. The material’s visual aids have clear titles or captions | 58% | 5% | 37% |
| 18. The material uses illustrations and photographs that are clear and uncluttered | 64% | 2% | 33% |
| 19. The material uses simple tables with short and clear row and column headings | 12% | 0% | 88% |
| **ACTIONABILITY** |  |  |  |
| 20. The material clearly identifies at least one action the user can take | 100% | 0% | 0% |
| 21. The material addresses the user directly when describing actions | 95% | 5% | 0% |
| 22. The material breaks down any action into manageable, explicit steps | 93% | 7% | 0% |
| 23. The material provides a tangible tool (e.g., menu planners, checklists) whenever it could help the user take action | 87% | 13% | 0% |
| 24. The material provides simple instructions or examples of how to perform calculations | 3% | 0% | 97% |
| 25. The material explains how to use the charts, graphs, tables, or diagrams to take actions | 2% | 0% | 98% |
| 26.  The material uses visual aids whenever they could make it easier to act on the instructions | 61% | 39% | 0% |
