# Supplementary material for: A health literacy analysis of the consumer-oriented COVID-19 information produced by ten state health departments
Source: J Med Libr Assoc. 2021 Jul 1;109(3):422–31. doi: 10.5195/jmla.2021.1165 (PMC8485956; doi:10.5195/jmla.2021.1165)
Supplement: Supplementary file 3 — Appendix C Index Item Scoring [file jmla-109-3-422-s03.docx]

Appendix C. Index Item Scoring
